# The effect of transtheoretical model-lead intervention for knee osteoarthritis in older adults: a cluster randomized trial

**DOI:** 10.1186/s13075-020-02222-y

**Published:** 2020-06-08

**Authors:** Limin Wang, Hongbo Chen, Han Lu, Yunlin Wang, Congying Liu, Xu Dong, Jieru Chen, Nan Liu, Fang Yu, Qiaoqin Wan, Shaomei Shang

**Affiliations:** 1 School of Nursing, Peking University, 38 Xueyuan Road, Hai Dian District, Beijing, China; 2 Department of Cardiology, Peking University Third Hospital, Beijing, China; 3 Department of Recovery, Peking University Third Hospital, Beijing, China; 4 School of Nursing, University of Minnesota, Minneapolis, USA

**Keywords:** Exercise adherence, Knee osteoarthritis, Intervention, Latent growth model, Symptom, Knee function

## Abstract

**Background:**

Knee osteoarthritis (KOA) is a common joint disease in people over 60 years old. Exercise therapy is one of the most effective non-pharmacological treatments for KOA, but low exercise adherence needs to be improved. The present study aimed to evaluate the effect of the transtheoretical model-lead home exercise intervention (TTM-HEI) program on exercise adherence, KOA symptoms, and knee function in older adults with KOA.

**Methods:**

A two-arm, superiority, assessor-blinded, cluster randomized trial was conducted. Community-dwelling older adults with KOA were recruited from 14 community centers in Beijing, China, via print and social media advertisements from April to October 2018. The present study lasted 48 weeks, with an intervention duration of 0–24 weeks and follow-up time of 24–48 weeks. The intervention was a two-stage and 24-week TTM-based exercise program, and the control group underwent a same-length exercise program guidance without any exercise adherence interventions. The primary outcome was exercise adherence to the prescribed home exercise program and was measured using an 11-point numerical (0 = not at all through and 10 = completely as instructed) self-rating scale at weeks 4, 12, 24, 36, and 48 after the program started. KOA symptoms (pain intensity and joint stiffness) were measured using the Western Ontario and McMaster Universities Osteoarthritis Index (WOMAC), and knee function (lower limb muscle strength and balance) was measured using the Five-Times-Sit-to-Stand Test (FTSST) and the Timed Up and Go Test (TUG) at baseline, week 24, and week 48. Latent growth model (GLM), repeated measures ANOVA, and independent *t* test were the main statistical tests used.

**Results:**

A total of 189 older adults (intervention group: *n* = 103, control group: *n* = 86) were enrolled. Differences of any outcome measures at baseline were not significant between groups. The growth rate of exercise adherence in the intervention group increased 2.175 units compared with that in the control group (unstandardized coefficient of slope on group B2 = 2.175, *p* < 0.001), and the intervention program maintained participants’ exercise adherence with 5.56 (SD = 1.00) compared with 3.16 (SD = 1.31) in the control group at week 48. In addition, the TTM-HEI program showed significant effects on relieving KOA symptoms and improving knee function.

**Conclusion:**

Over time, TTM-HEI could improve participants’ exercise adherence, KOA symptoms, and knee function.

**Trial registration:**

This study was approved by the ethics committee (IRB00001052-17066) in July 2017 and was registered at the Chinese Clinical Trial Registry (website: www.chictr.org.cn, registry number: ChiCTR1800015458).

## Background

Knee osteoarthritis (KOA) is a common joint disease in adults over 60 years old, with a prevalence of approximately 20–46% in China [1, 2] and 10–30% worldwide [3–6]. KOA mainly causes pain, joint stiffness, and loss of function. These physiological symptoms reduce the quality of life for adults with KOA and could cause negative emotions, such as depression and anxiety [7]. Exercise therapy is viewed as one of the primary non-pharmacologic treatments for KOA and is known to relieve pain and knee stiffness, enhance joint function, and improve patients’ quality of life [8, 9]. Exercise therapy is recommended to people with KOA and other chronic skeletal and musculoskeletal pain by physicians or rehabilitation trainers. However, poor exercise adherence has continually been a problem, especially when evaluated in the long term. The percentage of completed prescribed sessions (i.e., the number of completed home exercise sessions divided by the prescribed sessions) in an interventional trial for adults with KOA ranged from 37% in the physiotherapy group to 39% in the physiotherapy plus telephone coaching group during the 12–18-month follow-up [10]. Further, self-rated adherence to home exercise on an 11-point numerical rating scale (with 0 being not at all and 10 being completely as instructed) was only 3.6 and 3.8 during the same time frame in the two groups, respectively [10]. Low exercise adherence for older adults with KOA during 1 year or longer after the intervention has been reported in similar studies. The mean adherence rate (i.e., the number of completed home exercise sessions divided by the prescribed sessions) was reported as < 45% during the 9–12 months in supervised walking plus behavioral intervention group or in the supervised walking intervention group [11]. Additionally, the pooled results from three interventional studies conducted on adults with KOA showed that the mean adherence score (measured by self-rated 11-point scales) was 4.9 at week 42, 4.4 at week 52, and only 3.5 at week 78 [12].

Interventions to improve exercise adherence include health education, supervision and follow-up, goal setting, and booster sessions. (1) *Health education*: Nicolson et al. [13] reported that among many interventions to improve exercise adherence, health education is used most frequently and is also a form that patients are willing to accept. However, some studies have suggested that health education alone has little effect on the long-term maintenance of exercise behavior, despite being able to improve short-term exercise adherence [14]. (2) *Supervision and follow-up*: Gardner et al. [15] mentioned in their study that the supervision by physiotherapists can significantly increase the patient’s participation rate in the course and improve exercise adherence. Steele et al. [16] encouraged patients with chronic lung disease to adhere to home-based lung function rehabilitation training by telephone follow-up once a week. Telephonic follow-up included asking about exercise adherence and helping to solve problems encountered during exercise. The results showed that exercise adherence of patients improved within 6 months, but there was no significant improvement after 6 months. (3) *Setting goals*: The control theory of Carver [17] suggests that setting goals according to the actual situation of patients and updating the goals in time according to the changes of patients’ behavior are the core elements of stable healthy behavior. However, O’Brien et al. [18] attempted to improve exercise adherence in patients with KOA by setting goals. The results showed that the application of this measure alone can only improve the patient’s short-term exercise adherence, but has no effect on improving long-term adherence. (4) *Booster session*: Nicolson et al. [19] conducted a meta-analysis of the effects of interventions to improve exercise adherence. The results showed that booster sessions could significantly improve the patient’s exercise adherence, and the level of evidence was moderate. (5) Currently, interventions based on relevant theories are also generally used. Common theories used to formulate exercise adherence interventions included self-efficacy theory, planned behavior theory, social learning theory, and the transtheoretical model (TTM). Among them, TTM is increasingly used in intervention studies to improve exercise adherence because of its ability to formulate intervention strategies that match individual characteristics [20, 21].

Factors influencing exercise adherence in people with KOA were various [8], and no single strategy will be effective in overcoming all barriers to exercise participation in all people at all times [8, 19]. Exercise studies that integrate multiple intervention strategies—especially lead by theory—might effectively improve exercise adherence in patients with KOA in the long term [19, 22, 23]. The TTM [20, 21, 24], a stage theory about behavior change, provides a comprehensive framework and targeted measures for patients with different exercise psychological statuses or in diverse exercise stages [25]. In addition, essential factors that influence exercise adherence of patients, including psychological activity or behavioral strategies, are emphasized in TTM, thereby it might be effective to increase exercise adherence and improve patient outcomes [26, 27]. The TTM contains four concepts, including (1) stages of change, (2) process of change, (3) self-efficacy, and (4) decisional balance. The “stages of change” in the context of exercise can be described as follows: pre-contemplation (participant has no intention to start exercising within the next 6 months), contemplation (participant intends to start exercising within the next 6 months), preparation (participant plans to begin exercising within 1 month or is currently exercising irregularly), action (participant has been regularly exercising for < 6 months), and maintenance (participant has been regularly exercising for > 6 months) [28]. The “process of change” entails both covert and overt activities, including experiential and behavioral processes that participants employ as they progress through the stages of TTM [29]. Besides, this model provides important guidelines for intervention programs because the overall process consists of independent variables that individuals should adopt for progression and improvement. “Self-efficacy” refers to an individual’s confidence to change or to maintain a specific behavior throughout various situations. Self-efficacy usually increases as the individual advances throughout the “stages of change.” “Self-efficacy” impacts the association between the “process of change” and the “stages of change” [30]. “Decisional balance” consists of the individual’s perceived pros (i.e., benefits) and cons (i.e., costs) of changing their behavior over time. As an individual progresses through the stages of the TTM, the perceived benefits of the behavior increase, while the perceived barriers of the behavior decrease [31].

For the older adults who do not want to exercise at all, TTM provides intervention strategies targeted for the pre-contemplation stage and contemplation stage, which could increase the possibility of older adults to enter the action stage. For those who have started exercising but have not persisted regularly, TTM states that targeted measures (e.g., increasing social support, setting up stimuli to remind the exercise) could promote the individual to transition further into the action stage [32]. Recycling [24] is very common in older adults with KOA. For the older adults with osteoarthritis, 3 months after the end of exercise therapy is the fastest decline in compliance, and half of the older adults cannot adhere to exercise for ≥ 3 months [33]. This meant that individuals in the action stage (regularly exercising for < 6 months) would be backward to pre-action stages (pre-contemplation, contemplation, and preparation). Therefore, theoretically, the application of TTM could improve the exercise adherence of all older adults with KOA. However, studies using TTM for the intervention of older adults with KOA are limited, especially relating to the effect of TTM on individual’s exercise adherence long term.

Therefore, the aim of the present study was to evaluate the long-term effect of TTM-based home exercise intervention (TTM-HEI) on improving exercise adherence, KOA symptoms, and knee function in community-dwelling older adults with KOA.

## Methods

### Design

Our study was a two-arm, superiority, assessor-blinded, cluster randomized trial. The study lasted for 48 weeks, with an intervention time of 0 to 24 weeks and a follow-up period of 24 to 48 weeks. Participant characteristics were collected at baseline only. Exercise adherence of participants was collected at 4, 12, 24, 36, and 48 weeks. Secondary outcomes (KOA symptoms and knee function) were collected at 0, 24, and 48 weeks.

To avoid contamination within a community, randomization was performed at the community level instead of at the individual level. An independent researcher used the random number function in Excel to generate the randomization sequence. Study staff opened opaque envelopes with random numbers to obtain the community allocation.

Participants signed the informed consent and were informed of their assigned group and specific exercise intervention strategies. Therefore, participants were not blinded to the allocation of groups. Moreover, study staff were unmasked to the allocation of participants after community recruitment due to the differences in the exercise intervention programs. However, the assessor and the statistician were masked to the allocation of the participants.

### Sample and setting

Community-dwelling older adults with KOA were recruited from 14 community centers in Beijing via print and social media advertisements from April to October 2018. The inclusion criteria were as follows: age ≥ 60 years, had experienced knee pain on most days within the past month, scored their average knee pain over the past week between 3 and 7 on an 11-point numeric rating scale, and showed intact cognitive functioning, as indicated by a score of 8–10 on the 0–10-point Short Portable Mental Status Questionnaire [34]. The exclusion criteria were as follows: participants had undergone either a joint replacement or arthroscopic surgery on the affected side of the knee, had other lower limb surgery within the past 6 months, showed evidence of severe deformity of the lower limbs (e.g., knee varus or valgus), exhibited other health issues that could induce adverse events during home exercise (e.g., uncontrolled high blood pressure, myocardial infarction, cerebral infarction, unstable angina, arrhythmia, severe vision problems, or neurological dysfunction), or had other regular exercise habits (at least 3 days a week of no less than 30 min of exercise per day).

## Intervention

### Intervention group

#### General stage (weeks 0–2)

Participants in the intervention group entered the general stage after their baseline data were collected. The goals of this period for participants were to (i) correctly learn to perform home exercise, (ii) fully understand the basic knowledge of KOA and the benefits of exercise, and (iii) advance from a stage of pre-action (pre-contemplation, contemplation, and preparation) to a stage of action. Participants attended three 2-h group activities carried out by physiotherapists over 2 weeks. Each activity included an hour for group health education and another hour for exercise. The educational materials distributed to the participants included home exercise manuals and a printed version of the health education slides.

The exercise program was created based on literature review, clinical practice, and expert consultation. The exercise program had previously been proven to be effective to improve both symptoms and function of older adults with KOA [35]. It involved ten movements and was recommended to be practiced for 30–40 min per day on at least 3 days per week (Additional file 1).

Group health education was conducted by physiotherapists and was designed to increase participants’ awareness of exercise by explaining and discussing the severity of KOA and the benefits of exercise. It involves three parts that cover the concepts of (1) clinical signs, risk factors, treatment, and nursing care for KOA; (2) advantages and principles of exercise; and (3) final information related to routine daily care for KOA.

#### Stage-specific period (weeks 3–24)

In the stage-specific period, participants of each community were divided into two subgroups, including the pre-action stage subgroup and the action stage subgroup. Each subgroup had different intervention goals, and group activities were conducted separately. During this period, six group activities were held at weeks 4, 8, 12, 16, 20, and 24 (i.e., every 4 weeks) and each activity lasted about 2 h. The participants were required to participate in all six group activities. If a participant did not participate in a group activity for some reason, we supplemented the contents during the next group activity for him/her. Prior to every group activity, participants were re-assessed regarding their stage of change via phone or WeChat by research assistants and assigned to different subgroups, as necessary. The stage of change of the participants was assessed by the Questionnaire for Stage of Exercise Change. This 5-point scale developed by Marcus et al. [36] places the individuals in one of the following stages of change: pre-contemplation, contemplation, preparation, action, or maintenance. Therefore, members of each subgroup were assigned/reassigned based on the participants’ exercise conditions over the past 4 weeks, rather than being fixed into subgroups. Physiotherapists delivered TTM-based stage-matched interventions to the participants in each subgroup. Our study included a total of five physiotherapists who were the main interveners. They were responsible for exercise guidance and TTM interventions. They each had ≥ 5 years of musculoskeletal clinical experience and were given at least 2 h of training on home-based exercise programs. They also completed 6 h of training about intervention strategies and techniques based on TTM. A description of the core objectives, TTM-based strategies (mainly based on ten processes of change), and the recommended form of interventions at each stage are shown in (Additional file 2). At weeks 4 and 12, physiotherapists conducted two review sessions to ensure that participants continued to correctly perform home exercises.

### Control group

Participants in the control group received usual exercise guidance without any exercise adherence interventions. At baseline and weeks 1 and 2, physiotherapists carried out a total of three home exercise guidance sessions to ensure that participants were able to exercise at home correctly and safely, and to teach exercise precautions. Weeks 4 and 12 of the exercise review classes and the assessments were the same as in the intervention group. The content of the exercise guidance and the prescribed exercise type and intensity were exactly the same as in the intervention group.

### Measures

All study outcomes were collected during group activities at the corresponding time. Participant characteristics (at baseline), exercise adherence (at weeks 4, 12, 24, 36, and 48), and KOA symptoms (including pain intensity and joint stiffness at baseline, weeks 24 and 48) were collected through paper questionnaires. Knee functions (lower limb muscle strength and balance at baseline, weeks 24 and 48) were collected based on results of knee function tests.

### Participant characteristics

Baseline participant characteristics were obtained using a demographics questionnaire developed specifically for this study. The questionnaire included questions on age, sex, height and weight, marital status, educational level, occupation before retirement, residence, disease duration, comorbidities, and current drug use.

### Primary outcome measure

The primary outcome of the present study was exercise adherence of the participants. Exercise adherence was measured using an 11-point numeric rating scale (with 0 indicating not at all through and 10 indicating completely as instructed) at weeks 4, 12, 24, 36, and 48 [37]. The scale contains only one entry “Please rate your exercise adherence according to your performance with respect to the number of times of practice, quality of actions, and duration of each practice in the recent period.” If the participant wanted to evaluate his/her exercise adherence as 10 points, he/she were required to exercise 3–5 times a week for at least 30 min each time. The scale’s intra-class correlation coefficient was 0.77 when assessing exercise adherence among other populations with musculoskeletal disorders, which has proven to have an acceptable reliability [37].

### Secondary outcome measures

The secondary outcomes of our study included KOA symptoms, including pain intensity and joint stiffness, as well as knee function (lower limb muscle strength and balance), which were collected at baseline and weeks 24 and 48.

KOA-related pain intensity and joint stiffness were measured by the Western Ontario and McMaster Universities Osteoarthritis Index (WOMAC) [38]. It includes seven items related to pain and joint stiffness rated on a 0–4 Likert scale, where higher scores indicate greater pain and stiffness. The internal reliability of the Chinese version of the WOMAC, as measured by Cronbach’s *α*, is 0.67–0.82 across its two subscales. In addition, its test-retest reliability, based on the intra-class correlation coefficient, is 0.82–0.88 for its two subscales [39].

The adjusted total scores of pain intensity and joint stiffness ranged from 0 to 100, which were calculated from the raw ratings of the total scores as follows:

$$ \mathrm{Adjusted}\ \mathrm{score}\left(\mathrm{AS}\right)=\frac{\mathrm{Raw}\ \mathrm{rating}\left(\mathrm{RR}\right)}{\mathrm{The}\ \mathrm{total}\ \mathrm{score}\mathrm{s}}\times 100 $$

The muscle strength of the lower limbs was determined by the Five-Times-Sit-to-Stand Test (FTSST), which requires participants to rise from a chair and return to a seated position, with their arms folded across their chests five times as quickly as possible. Participants completed this exercise twice, with a 1-min rest period between each trial. The mean value of the two trials was used [40]. Participants’ balance was measured via the Timed Up and Go Test (TUG), which measures the time it takes for participants to rise from a standard height chair, walk 3 m, turn around, return to the chair, and sit down [41].

### Data collection

Community nurses recruited older adults diagnosed with KOA from 14 community centers. Doctors screened the participants according to the inclusion and exclusion criteria to determine the eligibility for participation. Next, participants signed the informed consent forms and completed the baseline assessments. Data were collected by three assessors and the assessors were blinded to the group assignments.

### Ethical considerations

Ethical approval was obtained from the Peking University Biomedical Ethics Committee (IRB00001052-17066) in July 2017. All participants voluntarily participated and could withdraw at any time without negative consequences. Each participant completed written informed consent. The data collected were anonymized and kept confidential and were used exclusively for the present study.

### Sample size

We used the two-sample *t* test power analysis for sample size calculation. The primary outcome was the difference in the exercise adherence score between the intervention group and control group at 24 weeks. Determining the mean difference (1.1) and SD (2) between the groups was based on the results of our pilot study and a relevant search on exercise interventions [12]. Power analysis was carried out with *α* = 0.05 and *β* = 0.2 and with the intervention and control groups having the same sample size. According to the Power Analysis and Sample Size software (PASS 2008, NCSS Corporation), 50 participants were required per group. Considering that the experimental design is a cluster randomized controlled trial (RCT), relevant factors within the community were taken into account and applied to the formula *N* = [1 + (*m* − 1) *ρ*] *n*, where *N* is the sample size of the cluster RCT, *n* is the sample size of the individual RCT, *m* is the number of individuals in the predicted community, and *ρ* is the intra-group correlation coefficient [42]. In this study, *m* = 15 was expected. According to the literature review [43, 44], we calculated *ρ* = 0.03 and *N* = 142; taking into account a probable 15% loss to follow-up, the total sample size was calculated as 168 cases with 84 cases in each group.

### Statistical analysis

We used the intention-to-treat analysis method. Data were analyzed using SPSS version 25.0 (IBM Corporation, Armonk, NY, USA). We considered a *p* value of ≤ 0.05 (two-sided) to indicate statistical significance. Descriptive statistics such as means and SDs, medians, interquartile ranges [IQR], frequencies, and percentages were calculated to indicate demographic and disease characteristics and outcome scores. Inferential statistics including an independent *t* test and repeated measurement ANOVA were also used to analyze the data.

The repeated measures ANOVA was achieved by a general linear model. We used Mauchly’s test of sphericity to check whether the data fit the statistical assumptions for conducting repeated measures ANOVA. When the data did not satisfy the spherical assumption (e.g., *p* < 0.05), the epsilon correction coefficient was used to correct the degree of freedom. When the results of repeated measures ANOVA showed that there was no interaction of group × time, the group main effect was used to judge the difference between groups. If there was an interaction of group × time, it meant that the data of the two groups had different trends with time. It was not possible to judge the difference between groups by repeated measures ANOVA, so we used an independent *t* test to compare the data at each time point to determine the difference between groups. In addition, we used one-way repeated measures ANOVA to test for differences among time points within the group.

In addition, for the primary outcome of exercise adherence, we used a combination of repeated measures ANOVA and latent growth model (LGM) for comprehensive analysis. Through repeated measures ANOVA, changes and differences in the mean of exercise adherence between the two groups during intervention and follow-up could be analyzed. LGM could further analyze the change rate of exercise adherence over time and quantify the difference in the growth rate in exercise adherence between the two groups, while considering the interindividual variation to better elucidate longitudinal stability and change [45] and evaluating the efficacy of TTM-HEI. This is because effective intervention programs could not only increase the population mean but also reduce interindividual variation. Thus, most participants in the intervention group could progress in a concentrated manner according to the expected stage of behavior change, thereby reducing the chance of regression and stagnation. The specific method is as follows:

There are two latent variables in LGM [46], which are labeled as the intercept and the slope, respectively. The intercept reflects the initial level of variables and is often restricted as an equal constant. The slope reflects the change rate of variables across time and is commonly restricted to a series of constants as linear, nonlinear, or freely estimated. In a freely estimated model, slope is restricted the first time and at the second or the last time with a value of 0 and a value of 1, respectively [46]. In LGM, the variation of two parameters could be analyzed by the variances or residual variances, representing the interindividual variation in the initial level and growth rate.

To better analyze the differences of exercise adherence between two groups over time, we constructed the models using two steps. First, we analyzed the effect of the groups on the initial level (intercept) and change rate (slope) by model 1 conducting group as a covariate (0: control group, 1: intervention group). Second, we conducted multiple-group analysis to analyze the characteristics of two groups by model 2 (control group) and model 3 (intervention group). In the three models, the value of 1 was restricted to all intercepts, and the value of 0 and value of 1 were restricted to the slope at the first time point (week 4) and the last time point (week 48), respectively, considering that the change rates of exercise adherence were unknown.

The parameters of LGM in the present study were estimated using maximum likelihood (ML) with 2000-replication bootstrapping to obtain stable and unbiased parameters [47]. Model fit indices were the chi-square to degree-of-freedom ratio (*χ*^2^/df; with values < 3 and < 5, indicating good and adequate fit, respectively), the standardized root mean square residual (SRMR; with value below 0.08 indicating reasonable fit), and the Comparative Fit Index (CFI; with values ≥ 0.90 indicating acceptable level for model fit) [48]. Model fit indices in all three models were all acceptable with | *χ*^2^/df = 4.001, SRMR = 0.032, and CFI = 0.966 in model 1; |*χ*^2^/df = 5.904, SRMR = 0.070, and CFI = 0.849 in model 2; and |*χ*^2^/df = 3.074, SRMR = 0.067, and CFI = 0.939 in model 3.

## Results

### Participant recruitment and follow-up

**Fig. 1:**
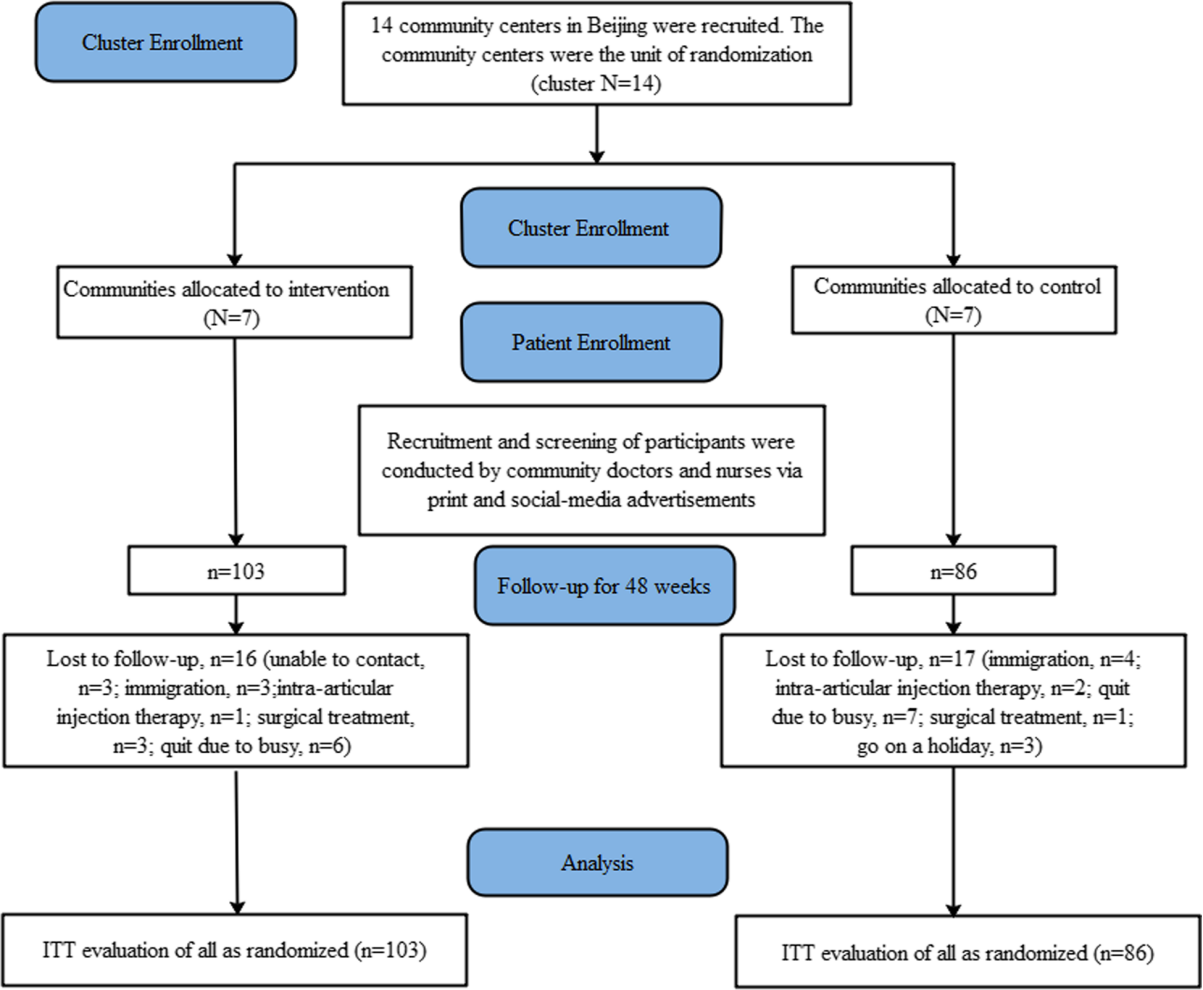
Flowchart of the study participants

Figure 1 shows the number of participants in the two groups at different time points. We recruited 280 participants from 14 community centers in Beijing, China; of these, 7 were randomly assigned to the intervention group and 7 to the control group. The patients were screened according to the inclusion criteria by the rehabilitation doctors, and 91 (32.5%) were excluded. The remaining 189 participants were randomly divided into two groups by community and included 103 in the intervention group and 86 in the control group. After 24 weeks, there were 161 participants (89 in the intervention group and 72 in the control group) that were retained. After 48 weeks, a total of 156 participants (87 in the intervention group and 69 in the control group) completed the collection of all outcomes. The total follow-up rate was 82.5%. A total of 16 participants were lost at follow-up in the intervention group and 17 in the control group. There was no significant difference in follow-up rates between the two groups (*χ*^2^ = 0.583, *p* = 0.445).

### Participant characteristics

The descriptive characteristics of the participants are presented in Table 1. The study involved 189 patients, including 103 patients in the intervention group, aged 60–82 years, with a mean age of 67.38 ± 7.79 years old. A total of 86 patients were in the control group, age 60–85 years, with a mean age 68.81 ± 6.74 years old. Most participants were female (92.6%), married (82.0%), with a high-school education (38.1%), and knee osteoarthritis in both knees (51.4%) and did not use a walker (94.2%). A small number of participants took analgesics (10.6%) and cartilage-protective drugs (15.3%) to relieve pain and other symptoms. The treatment groups were similar in demographics, clinical characteristics, and amount of therapy except the prevalence of diabetes (17.48% vs. 5.81% diabetics in the intervention and control groups, respectively, *χ*^2^ = 5.936, *p* = 0.015).

**Table 1:** The demographic characteristics of the recruited participants at baseline

Characteristic	Intervention (*n* = 103)		Control (*n* = 86)		*p* value
	*n*	(%)	*n*	(%)	
Age—mean (SD), years^‡^	67.38	(7.79)	68.81	(6.74)	0.182
Gender^†^					
Male	10	(9.7)	4	(4.7)	0.266
Female	93	(90.3)	82	(95.3)	
Body mass index—mean (SD), kg/m^2‡^	25.12	(3.58)	24.85	(3.05)	0.578
Symptom duration—mean (SD), years^‡^	7.54	(7.83)	7.08	(7.27)	0.680
Level of education^†^					0.215
Primary school or less	3	(2.9)	5	(5.8)	
Junior high school	26	(25.2)	29	(33.7)	
High school	39	(37.9)	33	(38.4)	
College graduate and above	35	(34.0)	19	(22.1)	
Marital status^†^					0.445
Single	16	(15.5)	17	(19.8)	
Married	87	(84.5)	69	(80.2)	
Number of affected knees^†^					0.259
One	54	(52.4)	38	(44.2)	
Two	49	(47.6)	48	(55.8)	
Uses a walker^†^					0.997
Yes	6	(5.8)	5	(5.8)	
No	97	(94.2)	81	(94.2)	
Comorbid conditions^†^					
Hypertension					0.077
Yes	44	(42.7)	26	(30.2)	
No	59	(57.3)	60	(69.8)	
Diabetes					0.015*
Yes	18	(17.5)	5	(5.8)	
No	85	(82.5)	81	(94.2)	
Coronary heart disease					0.085
Yes	12	(11.7)	4	(4.7)	
No	91	(88.4)	82	(95.4)	
Osteoporosis					0.578
Yes	32	(31.1)	30	(34.9)	
No	71	(68.9)	56	(65.1)	
Current drug use^†^					
Analgesics					0.669
Yes	10	(9.7)	10	(11.6)	
No	93	(90.3)	76	(88.4)	
Cartilage protection drugs					0.937
Yes	16	(15.5)	13	(15.1)	
No	87	(84.5)	73	(84.9)	

*SD* standard deviation

^†^Chi-square or Fisher’s exact tests were used

^‡^Independent samples *t* test was used

*p < 0.05 was considered statistically significant

### Exercise adherence

**Fig. 2:**
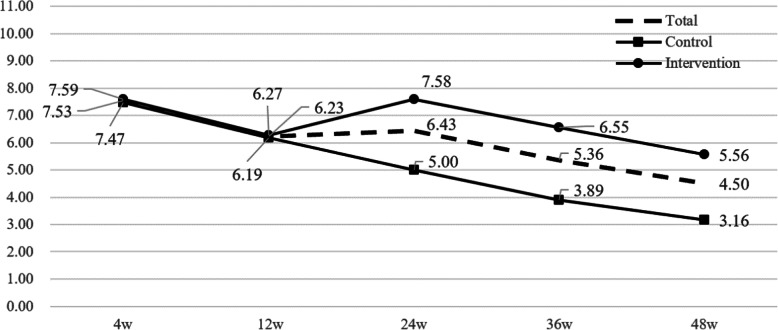
Exercise adherence scores in the intervention and control groups over 48 weeks

Table 2 and Fig. 2 show the exercise adherence scores in each group over 48 weeks. The scores in the intervention group and the control group are similar from weeks 4 to 12 with 7.59 (SD = 1.64) compared with 7.47 (SD = 2.24), respectively, at week 4, and 6.27 (SD = 1.86) compared with 6.19 (SD = 2.28), respectively, at week 12 (Fig. 2). From weeks 12 to 24, the exercise adherence score of the intervention group increased to 7.58 (SD = 1.29), whereas the control group continued to decline to 5.00 (SD = 1.53). Furthermore, from weeks 24 to 48, the exercise adherence scores of the intervention group and the control group both decreased to 5.56 (SD = 1.00) and 3.16 (SD = 1.31), respectively.

**Table 2:** Exercise adherence score over time by group

	Intervention (*n* = 103)	Control (*n* = 86)		
	Mean (SD)	Mean (SD)	*t*^†^	*p*
Week 4	7.59 ± 1.64	7.47 ± 2.24	0.450	0.653
Week 12	6.27 ± 1.86	6.19 ± 2.28	0.254	0.800
Week 24	7.58 ± 1.29	5.00 ± 1.53	11.646	< 0.001^#^
Week 36	6.55 ± 1.28	3.89 ± 1.53	12.043	< 0.001^#^
Week 48	5.56 ± 1.00	3.16 ± 1.31	12.999	< 0.001^#^
*F*^‡^	65.971	53.664		
*p*	< 0.001*	< 0.001*		

Exercise adherence was evaluated by the participants filling in the NRS (0 = not at all through 10 = completely as instructed)

*SD* standard deviation

^†^Independent *t* test was used

^‡^One-way repeated measurement ANOVA was used

*p < 0.05 was considered statistically significant

^#^*p* < *α*’ = 0.05/5 = 0.01 was considered statistically significant

Repeated measures ANOVA indicated that there was a significant difference in the trend of adherence scores between the two groups from baseline to week 48 (*F*_time × group_ = 18.400, *p*_time × grou*p*_ < 0.001, partial *η*^2^ = 0.328). Then, we used an independent *t* test to compare the adherence scores of each time point. The results showed that scores in the intervention group were significantly better than those in the control group at 24 weeks (*t* = 11.646, *p* < 0.001), 36 weeks (*t* = 12.043, *p* < 0.001), and 48 weeks (*t* = 12.999, *p* < 0.001). Also, there was no statistical difference in adherence scores at week 4 and week 12.

The largest absolute values of skewness and kurtosis statistics of any measurements for the total data and each group were 1.245 and 3.040, respectively, and all are within the accepted limits (absolute skewness < 2 and absolute kurtosis < 7 is acceptable) [49]. The results suggest that normality for LGM was met.

**Fig. 3:**
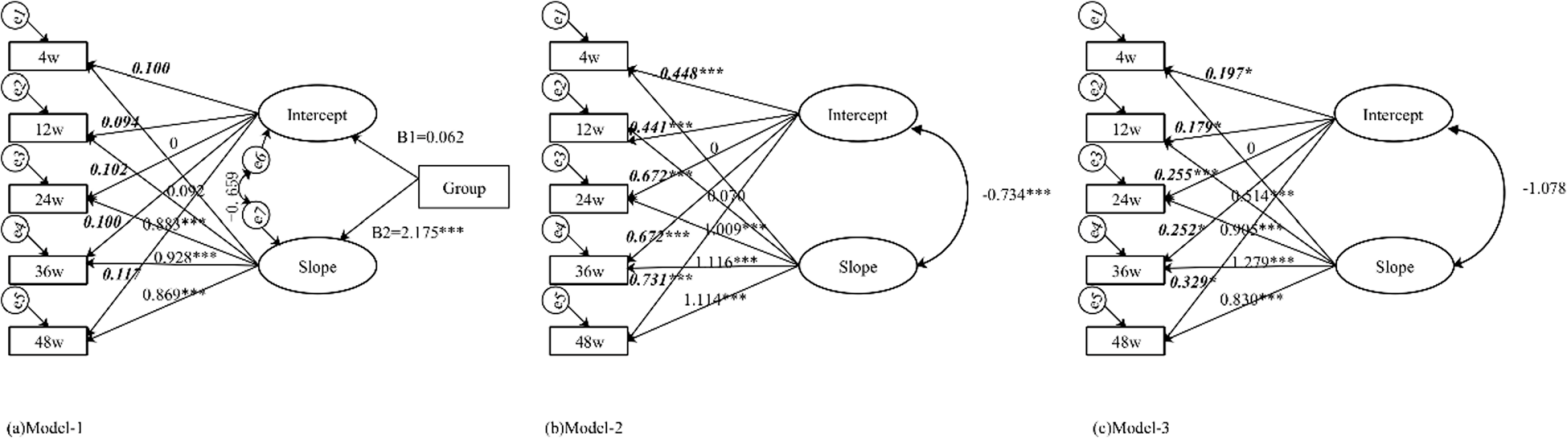
Main estimated parameter diagrams using the three models. **a** Model 1: conducting group as a covariate (0: control group, 1: intervention group). **b** Model 2: control group. **c** Model 3: intervention group. The path loading of the intercept is shown by bold italic font, and the path loadings of the slope are shown by regular front. ***p < 0.001, **p < 0.01, *p < 0.05

Figure 3 shows the main estimated parameters of the three LGM models. We observed no significant difference at the initial level of exercise adherence between the control and intervention groups shown by an insignificant unstandardized coefficient of intercept between the groups in model 1 (Fig. 3a, B1 = 0.062, SE = 0.076, *p* > 0.05). The growth rate of exercise adherence in the intervention group was greater (2.175 units) than that in the control group, with a positive and significant unstandardized coefficient of slope using model 1 (Fig. 3a, B2 = 2.175, SE = 0.118, *p* < 0.001). The negative mean of the slope in model 2 (mean = − 0.002, SE = 0.044, *p* > 0.05) and the positive mean of the slope in model 3 (mean = 0.024, SE = 0.056, *p* > 0.05) showed that exercise adherence may decline in the control group and slightly increase over time in the intervention group.

In addition, we analyzed the changes of exercise adherence over time within each group. Significant estimated slopes (Fig. 3b, c) and significant variances of slope (model 2 variances of slope = 2.304, SE = 0.381, *p* < 0.001 and model 3 variances of slope = 0.687, SE = 0.150, *p* < 0.001) showed the exercise adherence growth rate was observed in intraindividual variation and interindividual variation, but the interindividual variation of growth slope in the intervention group was much smaller than that in the control group. The negative correlation coefficients between the intercept and the slope (model 2: *R* = − 0.734, SE = 0.077, *p* < 0.001 and model 3: *R* = − 1.078, SE = 1.282, *p* > 0.05) showed that the exercise adherence growth rate declined with the initial level, but such inference should be careful for the intervention group because of the insignificant *p* values in model 3.

### Pain intensity and joint stiffness

From baseline to week 48, the intensity of pain in both groups decreased, but the rate of decline in the intervention group was significantly faster than that in the control group (*F*_time_ = 5.051, *p*_time_ = 0.008, partial *η*^2^ = 0.062; *F*_time × group_ = 3.301, *p*_time × group_ = 0.039, partial *η*^2^ = 0.041). There were no significant differences in pain intensity at baseline between the two groups. However, pain intensity in the intervention group was significantly lower than that in the control group at week 24 (*t* = − 2.793, *p* = 0.006) and week 48 (*t* = − 2.550, *p* = 0.012). Joint stiffness improved in both groups during the study, but the exercise in the intervention group was more effective than that in the control group (*F*_time_ = 3.813, *p*_time × group_ = 0.024, partial *η*^2^ = 0.047; week 24, *t* = − 3.376, *p =* 0.001; week 48, *t* = − 2.611, *p =* 0.010). An intra-group comparison revealed that over three time points knee stiffness in the intervention group was significantly different over the 48-week time period compared with the control group (*F* = 13.374, *p* < 0.001, partial *η*^2^ = 0.239) (Table 3; Additional file 3; Fig. 1a, b).

**Table 3:** Secondary outcome measures over time in the control and intervention groups

	Intervention (*n* = 103)	Control (*n* = 86)		
	Mean (SD)	Mean (SD)	*t*^†^	*p*
Pain intensity^#^				
Baseline	24.37 ± 20.31	24.42 ± 19.65	− 0.017	0.986
Week 24	16.18 ± 15.94	23.47 ± 17.11	− 2.793	0.006^#^
Week 48	13.62 ± 11.28	19.64 ± 16.83	− 2.550	0.012^#^
*F*^‡^	7.072	2.085		
*p*	0.001*	0.131		
Joint stiffness^#^				
Baseline	24.03 ± 24.73	25.00 ± 25.37	− 0.266	0.791
Week 24	10.53 ± 12.49	19.62 ± 19.88	− 3.376	0.001^#^
Week 48	9.77 ± 14.19	17.57 ± 21.36	− 2.611	0.010^#^
*F*^‡^	13.374	0.945		
*p*	< 0.001*	0.394		
Lower limb muscle strength				
Baseline	12.03 ± 5.09	12.27 ± 4.29	− 0.350	0.727
Week 24	9.61 ± 2.43	11.34 ± 3.66	− 3.583	< 0.001^#^
Week 48	10.29 ± 3.70	11.06 ± 2.79	− 1.442	0.151
*F*^‡^	19.441	3.565		
*p*	< 0.001*	0.034*		
Balance				
Baseline	2.95 ± 0.29	2.03 ± 0.22	− 0.775	0.439
Week 24	1.26 ± 0.13	1.60 ± 0.19	− 4.747	< 0.001^#^
Week 48	1.11 ± 0.12	1.22 ± 0.15	− 2.576	0.011^#^
*F*^‡^	13.847	6.687		
*p*	< 0.001*	0.002*		

*SD* standard deviation

^†^Independent *t* test was used

^‡^One-way repeated measurement ANOVA was used

*p < 0.05 was considered statistically significant

^#^The score range for pain intensity and joint stiffness is 0–100; *p* < *α*’ = 0.05/3 ≈ 0.017 was considered statistically significant

### Lower limb muscle strength and balance

From baseline to week 48, the lower limb muscle strength and balance increased in the control group, but first increased and then decreased in the intervention group (Table 3). Repeated measurement ANOVA indicated that the lower limb muscle strength increased in the intervention group compared with the control group from baseline to week 48 (*F*_time_ = 16.853, *p*_time_ < 0.001, partial *η*^2^ = 0.181; *F*_time × group_ = 5.782, *p*_time × group_ = 0.004, partial *η*^2^ = 0.070). There were no statistical differences at baseline (*t* = − 0.350, *p* = 0.727) and at week 48 (*t* = − 1.442, *p* = 0.151) between the two groups; however, lower limb muscle strength in the intervention group was significantly higher than that in the control group at week 24 (*t* = − 3.583, *p* < 0.001). Similarly, from baseline to week 48, improvements in balance in the intervention group were more significant compared with the control group (*F*_time_ = 12.970, *p*_time_ < 0.001, partial *η*^2^ = 0.146; *F*_time × group_ = 5.575, *p*_time × group_ = 0.005, partial *η*^2^ = 0.068; week 24, *t* = − 4.747, *p <* 0.010; week 48, *t* = − 2576, *p =* 0.011) (Table 3; Additional file 3; Fig. 1c, d).

## Discussion

Our study showed that TTM-HEI could significantly improve exercise adherence, KOA symptoms (both pain and joint stiffness), and physical function (muscle strength of the lower limbs and balance) in the long term compared with normal exercise guidance. The rates of loss to follow-up were 15.5% and 19.8% in the intervention and control groups, respectively.

During the 48-week follow-up period, the growth rate of exercise adherence in the intervention group was greater (2.175 units) than that in the control group. In addition, from the perspective of interindividual differences, the slope variation of the intervention group was 0.687 ± 0.150, while that of the control group was 2.304 ± 0.381, indicating that the interindividual difference in the intervention group was smaller than that in the control group, and exercise adherence was more consistent and stable. Besides, although there was no difference in adherence between the TTM-HEI groups in short term (i.e., the first 12 weeks from the start of the intervention), the difference was significant in the middle and long term. The possible reasons may be as follows: (i) The TTM intervention program could improve the participation rate of the population. Through the use of some targeted processes of change, participants in the pre-contemplation stage and contemplation stage are fully aware of the benefits of exercise and are willing to establish exercise behaviors, thereby transitioning to the action stage. Traditional exercise instruction would not be wide reaching, especially to this population. (ii) TTM intervention program could target participants who are exercising by helping them overcome obstacles, resolving conflicting emotions, and encouraging them to continue to exercise. (iii) The TTM intervention program could encourage participants who have recycled back into earlier stages to resume exercise. During the intervention, there was a certain phenomenon of recycling in the intervention group. As is now well known, most people taking action to change behavior do not successfully maintain their gains on their first attempt [24]. When the physiotherapists found that participants had a recycling, they first assessed which stage they had backward to, and then understood the reason for the recycling. According to the specific reasons, the physiotherapists implemented intervention strategies to match the current stage of change and encouraged recycled participants to resume exercise. Thereby, TTM-HEI could reduce exercise withdrawal rate and further improve exercise adherence. (iv) The TTM intervention program focused on producing change at the level of the participants’ experience and at the level of their environment. At the level of the participants’ experience, TTM-HEI not only raised participants’ consciousness about the benefit of regular exercise for themselves but also inspired them to think about the benefits to their families and friends. Sharing experience, communication, and consultation could improve their confidence to change or maintain exercise throughout various situations (i.e., self-efficacy). At the level of their environment, TTM-HEI could guide participants to make use of social relationship and resources to help them overcome the obstacles of exercise and create a suitable environment, such as making a commitment to exercise and transforming home environment. The 10 processes of change in TTM also emphasized the possible variables influencing the behavior change. The TTM-HEI integrated the processes of change and stages and used multiple techniques, such as self-monitoring by maintaining an exercise diary and interpreting experience by group activities based on TTM and literature, to motivate participants’ regular exercise. (v) To increase the information available to participants and make them the most effective adherence to exercise, the TTM-HEI has adopted group activities in the intervention. Group activities were important to deliver information and stimulate motivation of adherence. In this study, we found that setting a “successful example” was an effective method to motivate participants to start exercising or exercise regularly. During the intervention, there was a phenomenon that participants always compared their adherence to the adherence of “successful example.” The “successful example” was one with high adherence or good improvement of knee function or symptoms and was selected by either researchers or the participants by communication. Peers who have achieved symptom relief and function improvement through exercise could motivate participants to increase confidence and become better exercisers. Hence, TTM-HEI could help participants maintain adherence to regular exercise.

Although TTM-HEI was effective to improve exercise adherence among older adults with KOA compared to the control group in the long term, adherence in the TTM-HEI group declined over time since the end of the intervention. Under the absence of any intervention or supervision, exercise adherence of people with KOA generally declines over time. Bennell et al. [10] showed that telephone coaching intervention enhanced adherence to home-based exercise (either the percentage of prescribed sessions completed or self-rated adherence to prescribed home exercise using the 11-point NRS) for patients with KOA during the 6-month intervention phase. However, the difference between groups in the two measurements of adherence was insignificant during the follow-up phase, and exercise adherence in both the intervention and control groups showed a decline after the end of the intervention [10]. Such decline in the long term could be found in similar studies for KOA [11, 12, 50]. In this study, although the exercise adherence of the intervention group also decreased over time, the decline rate was significantly slower than that of the control group. During the long-term follow-up, the adherence of the intervention group was still maintained at a relatively high level, and this level of adherence was sufficient to maintain the improvement of KOA symptoms and functional improvement in the intervention group. We believe that this could reflect the positive effect of the TTM program on exercise adherence. Besides, we are interested in the effect of TTM-HEI on exercise adherence and other outcomes in the long term, i.e., the differences in outcomes between groups during the follow-up phase. So we added none any intervention in follow-up time frame. Our results verified that TTM-HEI could maintain exercise adherence at a relatively high level in the long term compared to the control group. In practice, when applying TTM-HEI, proper booster sessions in the follow-up phase might be necessary to avoid such decline and maintain a higher level of adherence.

When investigating health behaviors, theories are helpful to describe and understand processes, gain knowledge, and accumulate evidence [22]. Hence, intervention based on behavior change theory might be effective to form and promote exercise behavior among adults with KOA. Review of studies aimed to improve exercise adherence among adults with KOA showed that only a few studies explicitly referred to the use of behavior change theory or other conceptual frameworks in developing intervention [11, 19, 51]. Because the content, intensity, measurements (method or time), and follow-up interval of intervention varied across studies, we could not directly compare the effect of TTM-HEI on exercise adherence with that in other studies. TTM clearly provides guidance for considering multiple factors that potentially influence adherence to exercise and techniques to overcoming these factors. By assessing stages of change, researchers could understand the process of exercising behavior change and make intervention targeted. With respect to the long-term effect of intervention on adherence, we conclude that the effect in TTM-HEI study was not inferior to that reported in existing studies.

In addition, we found that KOA symptoms (pain intensity and joint stiffness) and knee function (lower limb muscle strength and balance) improved from 0 to 48 weeks in both the intervention and control groups; however, the degree of improvement in the intervention group was significantly larger than that in the control group. We found that from 0 to 24 weeks, knee function, muscle strength, and balance in the intervention group improved rapidly, but the improvement rate slowed or decreased slightly from 24 to 48. However, the improvement in the control group was at a slower rate than that in the intervention group. Therefore, given that both groups performed the same exercise program, the changes observed between the intervention group and the control group were possibly caused by differences in exercise adherence. Our results corroborated previous studies, which showed the direct positive relationship between exercise adherence and exercise-related outcomes [52, 53]. A previous study showed that the lack of exercise compliance was the main impediment to the positive expected outcomes of exercise intervention in KOA patients [54]. Therefore, improving exercise compliance is the key to the successful long-term effects of exercise intervention.

Our study has several limitations. First, neither participants nor physiotherapists were blinded to group allocation, possibly resulting in an overestimation of the effects of the exercise intervention. Second, due to limited resources, we only included participants from the Beijing urban area. It is unclear if TTM-HEI is effective for additional types of older adults with KOA, for example, those from a rural location. Third, since we recruited participants via print and social media, those who were already interested in being active and caring for themselves were more likely to participate. This may have increased the selection bias. Fourth, perhaps because of the more active participation of women in community activities, most of the participants included in this study were women, which may have led to a degree of gender bias when interpreting results. Last, we applied a self-rating scale to evaluate exercise adherence, and some patients may have recall bias which could decrease accuracy. Future studies are required to introduce objective outcomes to assess exercise adherence.

## Conclusion

This assessor-blinded, cluster randomized study showed that TTM-HEI program can improve exercise adherence, KOA symptoms, and physical function in older adults with KOA in the long term. The TTM-HEI program can be applied in community centers to enhance exercise adherence and achieve good practical results.

## Supplementary information

**Additional file 1: Table S1.** Details of home-based exercises outcome measures over time according to group.

**Additional file 2: Table S2.** Guidelines of goals, processes, and interventions.

**Additional file 3: Figure S1.** Group differences in secondary outcomes over time.

**Additional file 4: Table S3.** and **Table S4.** Intervention implementation program.

## Data Availability

The datasets used and analyzed in the current study are available from the corresponding author upon reasonable request.

## Abbreviations

KOA: Knee osteoarthritis
TTM: The transtheoretical model
TTM-HEI: Transtheoretical model-lead home exercise intervention
LGM: Latent growth model
