# Supplementary material for: The effect of transtheoretical model-lead intervention for knee osteoarthritis in older adults: a cluster randomized trial
Source: Arthritis Res Ther. 2020 Jun 8;22:134. doi: 10.1186/s13075-020-02222-y (PMC7278156; doi:10.1186/s13075-020-02222-y)
Supplement: Supplementary file 1 — Additional file 1: Table S1. Details of home-based exercises outcome measures over time according to group. [file 13075_2020_2222_MOESM1_ESM.docx]

**Additional file 1**

Table S1. Details of home-based exercises

| Exercises | Details |
| --- | --- |
| Isometric contractions of the quadriceps | 1. Sitting or lying down, legs relaxing;  2. Tight the thigh muscles on one side with maximum strength, keep it for 5 seconds, and relax for 2 seconds. Repeat 10  times for 1 group and practice 10 groups in succession;  3. Relax this leg and repeat the above action on the other side;  4. Exercise alternately 3 to 5 times with both legs. |
| Supine straight-leg lifts | 1. Lie on the back, stretch knees;  2. One leg is flexed to support the bed surface, the other leg is raised to the heel, about 20 cm away from the bed, held for 5  seconds, put down for 5 seconds, repeat 10 times；  3. Relax this leg and repeat the above action on the other side；  4. Exercise alternately 3 to 5 times with both legs. |
| Leg lifts in the prone position | 1. Lie face down, stretch knees;  2. Lift one leg back to the toe, about 20 cm away from the bed, held for 5 seconds, put down for 5 seconds, repeat 10 times;  3. Relax this leg and repeat the above action on the other side;  4. Exercise alternately 3 to 5 times with both legs. |
| Passive knee flexion | 1. Sit on the bed;  2. Hold your hands on one side of the ankle, slowly and forcefully hold the leg to the chest to maximize knee flexion, keep  60 seconds;  3. Relax this leg and repeat the above action on the other side;  4. Exercise alternately 2 to 3 times with both legs. |
| Exercises | Details |
| Passive knee extension | 1. Sit on the bed;  2. Put one side of the foot pad 8~10 cm high;  3. Apply light weight to the raised knee joint or apply proper pressure by hand for 60 seconds;  4. Relax this leg and repeat the above action on the other side;  5. Exercise alternately 2 to 3 times with both legs. |
| Resistance knee extension | 1. Sit on the chair or at the bed, tie a 1kg sandbag to the ankle, keep the upper body straight;  2. Do not move the thighs, lift your calves until the knees are fully extended, hold for 5 seconds, rest your legs for 5  seconds, repeat 10 times;  3. Relax this leg and repeat the above action on the other side;  4. Exercise alternately 2 to 3 times with both legs. |
| Resistance knee flexion | 1. Standing up, tie a 1kg weight sandbag to the ankle joint, and support the upper edge of the chair;  2. Stand on one leg and pull the calf back to the other leg, flexing the knee as much as possible while keeping the thigh  perpendicular to the ground. Hold for 5 seconds, put your legs down for 5 seconds, repeat 10 times  3. Relax this leg and repeat the above action on the other side;  4. Exercise alternately 2 to 3 times with both legs. |
| Shifting the center of gravity (left and right) | 1. Stand up and support a table with a height of 70~80 cm and open the feet;  2. Keep your knees upright, slowly move the center of gravity to the left, and gradually lower your right heel;  3. Keep your knees upright, slowly move the center of gravity to the right, and gradually lower your left heel;  4. Repeat the above action for 3 minutes. |
| Exercises | Details |
| Shifting the center of gravity (forwards and backwards) | 1. Stand up and support a table with a height of 70~80 cm and take one step forward on one side;  2. Keep the knees upright, slowly move the center of gravity forward, and the heel of the hind foot gradually leaves the  ground;  3. Keep the knees upright, slowly move the center of gravity backwards, and the forefoot gradually leaves the ground;  4. Repeat the above action for 3 minutes. |
