# Supplementary material for: The effect of transtheoretical model-lead intervention for knee osteoarthritis in older adults: a cluster randomized trial
Source: Arthritis Res Ther. 2020 Jun 8;22:134. doi: 10.1186/s13075-020-02222-y (PMC7278156; doi:10.1186/s13075-020-02222-y)
Supplement: Supplementary file 2 — Additional file 2: Table S2. Guidelines of goals, processes, and interventions. [file 13075_2020_2222_MOESM2_ESM.docx]

Table S2. Guidelines of goals, processes and interventions for changing exercise behavior in each stage

| Stage | Goal | Processes of Change | Core of Interventions | Recommended Form of Interventions |
| --- | --- | --- | --- | --- |
| Precontemplation | Increase awareness of the need to exercise | Consciousness raising | Provide information on the benefits of exercise | - Group health education - One-on-one consultation |
|  |  | Dramatic relief | Trigger the patient's negative emotions and concerns about the dangers of not exercising | - Group health education(Introduction of cases with adverse outcomes) - Patients communication |
|  |  | Self-re-evaluation | Inspire patients to think about what positive changes will happen if they keep exercising | - One-on-one consultation - Experience sharing of exercise role model |
|  |  | Environmental re-evaluation | Inspire patients to think about what benefits they will bring to their families and friends if they keep exercising | - One-on-one consultation - Experience sharing of exercise role model |
| Contemplation | Increase motivation and confidence in exercising | Consciousness raising | Continue to provide information about the benefits of exercise and help reduce the obstacles to exercise | - Group health education - One-on-one consultation |
|  |  | Dramatic relief | Trigger the patient's negative emotions and concerns about the dangers of not exercising | - Group health education(Introduction of cases with adverse outcomes) - Patient communication |
|  |  | Self-re-evaluation | Inspire patients to think about what positive changes will happen if they keep exercising | - One-on-one consultation - Experience sharing of exercise role model |
|  |  | Environmental re-evaluation | Inspire patients to think about what benefits they will bring to their families and friends if they keep exercising | - One-on-one consultation - Experience sharing of exercise role model |
|  |  | Social liberation | Inspire patients to realize that some changes raise the availability of exercise in society | - One-on-one consultation |
| Stage | Goal | Processes of Change | Core of Interventions | Recommended Form of Interventions |
| Preparation | Negotiate a plan for exercising | Self-re-evaluation | Help patients create themselves the image of new exercisers | - One-on-one consultation |
|  |  | Helping relationships | Urge patients to seek help and encouragement from family and friends | - One-on-one consultation |
|  |  | Self-liberation | Help patients make a commitment to rehabilitation and announce to their family and friends | - Sign the exercise commitment |
|  |  | Stimulus control | Encourage patients to transform their home environment to remind themselves to exercise | - One-on-one consultation |
|  |  | Counterconditioning | Teach patients to use knee-friendly exercise instead of harmful exercises for knee joints | - Group health education - One-on-one consultation |
| Action | Reaffirm commitment and follow up | Self-liberation | Help patients make a commitment to exercise and announce to family and friends | - Sign the exercise commitment |
|  |  | Reinforcement management | Reward patients who have achieved the exercising goals | - Oral reward - Material reward |
|  |  | Counterconditioning | Help patients build a series of positive reactions to replace the occasional negative thoughts and concerns in exercising | - One-on-one consultation |
|  |  | Helping relationships | Help patients join the exercising group and urge each other to adhere to exercise | - Group experience exchange meeting |
|  |  | Stimulus control | Help patients set reminders to create an environment conducive to exercise | - One-on-one consultation - Disbursement and recycling of exercise records |
| Stage | Goal | Processes of Change | Core of Interventions | Recommended Form of Interventions |
| Maintenance | Solve the obstacles to adhere to exercise and prevent return | Counterconditioning | Help patients build a series of positive reactions instead of occasional suspicions about whether they can persist in exercising or not | - One-on-one consultation |
|  |  | Stimulus control | Help patients set reminders to create an environment conducive to exercise | - One-on-one consultation - Disbursement and recycling of exercise records |
|  |  | Helping relationships | Help patients join the exercising group and urge each other to adhere to exercise | - Group experience exchange meeting |
|  |  | Self-re-evaluation | Inspire patients to revisit themselves as an "exercise" | - One-on-one consultation |
|  |  | Reinforcement management | Reward patients who have achieved the exercising goals | - Oral reward - Material reward |
