# Supplementary material for: The effect of transtheoretical model-lead intervention for knee osteoarthritis in older adults: a cluster randomized trial
Source: Arthritis Res Ther. 2020 Jun 8;22:134. doi: 10.1186/s13075-020-02222-y (PMC7278156; doi:10.1186/s13075-020-02222-y)
Supplement: Supplementary file 3 — Additional file 3: Figure S1. Group differences in secondary outcomes over time. [file 13075_2020_2222_MOESM3_ESM.docx]

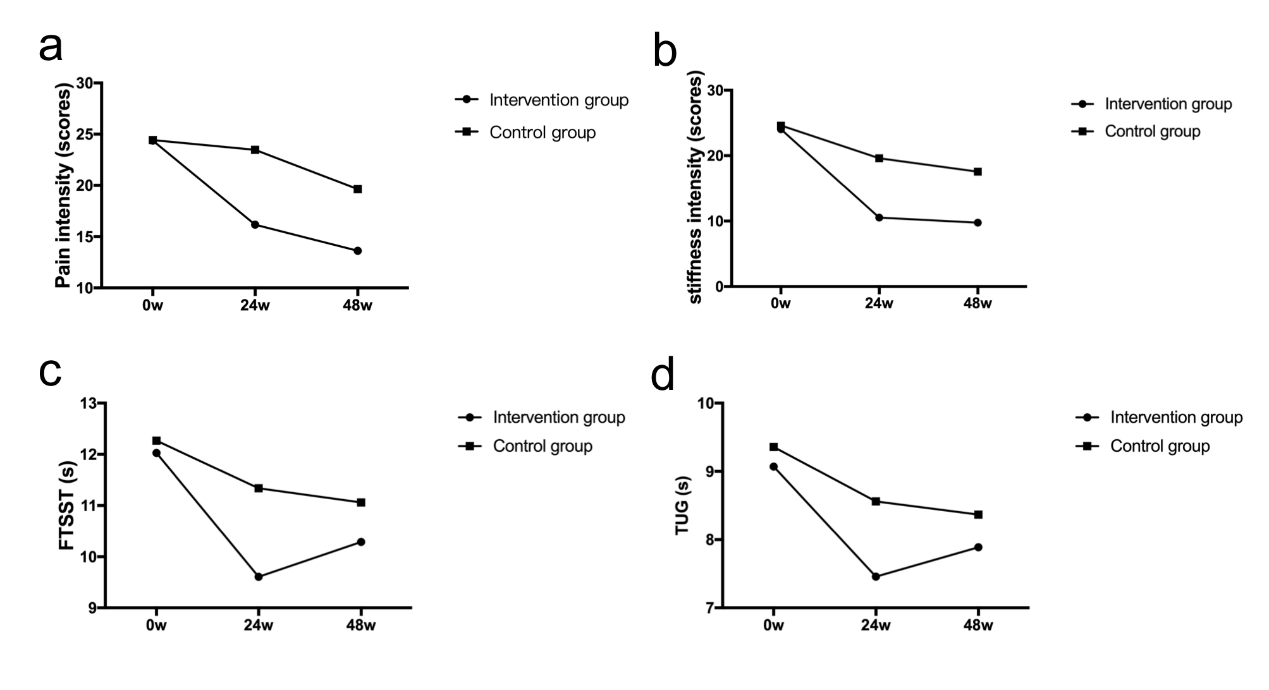


Figure S1. Group differences in secondary outcomes over time

a. Pain intensity of both groups over 48 weeks.

b. Joint stiffness of both groups over 48 weeks.

c. Lower limb muscle strength (the Five-Times-Sit-to-Stand Test (FTSST)) of both groups over 48 weeks.

d. Balance function (the Timed Up and Go test (TUG)) of both groups over 48 weeks.
