# Supplementary material for: The effect of transtheoretical model-lead intervention for knee osteoarthritis in older adults: a cluster randomized trial
Source: Arthritis Res Ther. 2020 Jun 8;22:134. doi: 10.1186/s13075-020-02222-y (PMC7278156; doi:10.1186/s13075-020-02222-y)
Supplement: Supplementary file 4 — Additional file 4: Table S3. and Table S4. Intervention implementation program. [file 13075_2020_2222_MOESM4_ESM.docx]

Table S3. Intervention implementation program based on TTM for general stage (Week 0~2)

| **Time** | **Form of Interventions** | **Specific Contents** |
| --- | --- | --- |
| Week 0  (Baseline) | Group health education I | Explain "what is KOA" and "exercise-related knowledge" t**o make participants aware of the benefits of exercise and the hazards of not performing exercise.** |
|  | Home exercise guidance I | Explain the composition of the entire exercise program and exercise precautions, **and emphasize the benefits of home exercise and the importance of home exercise safety.** Lecture on the "balance training and range of motion training module" in the exercise program. |
|  | One-on-one consultation | Answer the participants’ questions, assess the participants’ stage of change, and understand the participants’ psychological state of exercise. |
|  | Issuing exercise records I | Issue exercise records I (corresponding to the content of home exercise guidance I), explain how to fill out the exercise records, and ask the participants to complete and submit them during the next group activity **as a means of exercise monitoring and follow-up.** |
| Week 1 | Group health education II | Explain "KOA disease risk factors" and "pharmacotherapy related knowledge". Through the study of disease risk factors, remind the participants how to avoid the factors that can aggravate or induce KOA in daily life, and realize that many risk factors can be avoided, **reduce the negative awareness and negative emotions of some participants facing KOA.** Through the explanation of drug treatment related knowledge, the participants understand the basic effects and adverse reactions of KOA commonly used drugs, **become more aware of the economy, convenience and safety of exercise, and inspire them to start to establish exercise habits.** |
|  | Home exercise guidance II | Review the movements and precautions of home exercise guidance I, and continue to explain the movements and precautions of home exercise guidance II. **Emphasize the benefits of home movement and the importance of home movement safety.** |
|  | Retrieving exercise records I  Issuing exercise records II | Retrieve the exercise records I, and issue the exercise records II (corresponding to the content of the home exercise guidance II), and ask the participants to complete and submit it during the next group activity **as a means of exercise monitoring and follow-up.** |

| **Time** | **Form of Interventions** | **Specific Contents** |
| --- | --- | --- |
| Week 1 | Group experience exchange meeting | Guide the participants to exchange their experiences and gains and jointly solve the obstacles and difficulties encountered during the exercise. **Encourage them to establish regular exercise habits as soon as possible.** |
|  | Issuing evaluation reports | Based on the 0-week knee function evaluation results, an individualized knee function evaluation report was prepared for each participant. The report includes the individual's knee function, the average knee function in the community, and the individual's ranking in the community. After the participants understand their basic level of knee function, explain the meaning of various outcomes to them, **and encourage them to adhere to regular exercise, the indicators of knee function are expected to become better.** |
| Week 2 | Group health education III | Explain "KOA preventive health knowledge" to help participants establish healthy lifestyles to protect the knee in daily life. And encourage participants to make appropriate changes to their home environment to create a safe and suitable environment for home exercise. |
|  | Home exercise guidance III | Review the movements and precautions of Home exercise guidance I and II, and continue to explain the movements and precautions of Home exercise guidance III. **Emphasize the benefits of home movement and the importance of home movement safety.** |
|  | Group experience exchange meeting | Guide the participants to exchange their experiences and gains and jointly solve the obstacles and difficulties encountered during the exercise. **Encourage them to establish regular exercise habits as soon as possible.** |
|  | Retrieving exercise records II  Issuing exercise records III | Retrieve the exercise records II, and issue the exercise records III (corresponding to the action content of the home exercise guidance III), and asking the participants to complete and submit it during the next group activity **as a means of exercise monitoring and follow-up.** |
|  | Sign the exercise commitment | Distribute the "exercise goal commitments" to the participants, ask the participants to make a commitment, and encourage them to post the commitments in an obvious place at home, and **invite family members to supervise their adherence to exercise.** |

Table S4. Intervention implementation program based on TTM for stage-specific period (weeks 3-24)

| **Time** | **Form of Interventions** | **Specific Contents** |
| --- | --- | --- |
| Pre-action stage subgroup |  |  |
| 4^th^, 8^th^, 12^th^, 16^th^, 20^th^, 24^th^ week | Experience sharing of exercise role model | Ask the participants who exercise well to introduce the experience of adhering to exercise, which include the method of integrating exercise into daily life, and how to overcome it when encountering obstacles to exercise. **Highlight the benefits of exercise, as well as positive changes in physical and psychological aspects, and encourage members of the pre-action stage subgroup to start regular exercise as soon as possible and enter the action stage.** |
|  | Issuing evaluation report | Create individualized knee function evaluation report for each participant. The report includes the individual's knee function and the change of the previous evaluation results, the average knee function of the community, and the individual's ranking in the community. **Make them understand their knee function, and timely distribute to them the evaluation report of the more advanced members of the action stage subgroup, so that they can realize the benefits of exercise and start to exercise.** |
|  | Group experience exchange meeting | The members of this subgroup discuss the benefits and obstacles of adhering to the exercise, **find out why they have not started exercising, discuss ways to overcome the obstacles, and urge each other to start exercise.** |
|  | One-on-one consultation | Fully understand the reason why the participant did not enter the action stage, and evaluate the specific stage of change at present **to help them purposefully solve the current obstacles to adherence to the exercise.** |
| 6^th^, 10^th^, 14^th^, 18^th^, 22^th^ week | One-on-one consultation | Carried out by WeChat or telephone, the specific content is the same as above. |

| **Time** | **Form of Interventions** | **Specific Contents** |
| --- | --- | --- |
| Action stage subgroup |  |  |
| 4^th^, 8^th^, 12^th^, 16^th^, 20^th^, 24^th^ week | Group supplementary knowledge lecture | Collect participants opinions and give supplementary lectures on other knowledge that participants want to know. |
|  | Disbursement and recycling of exercise records | The exercise records III are issued and recovered **as a means of long-term exercise monitoring and follow-up.** |
|  | Issuing evaluation report | Create individualized knee function evaluation report for each participant. The report includes the individual's knee function and the change of the previous evaluation results, the average knee function of the community, and the individual's ranking in the community. Let the participants understand their knee function and changes, and encourage participants to compare with the previous knee function evaluation results. **The progressives gain the confidence to continue exercise, and the regressors find out the reasons and improve them.** |
|  | Group experience exchange meeting | The participants exchange their experiences and gains during the exercise, and jointly solve the obstacles and difficulties during the exercise. **Continue to exercise to prevent return.** |
|  | Oral / Material reward | The participants who have recently adhered to exercise are given verbal and material rewards and **encouraged to continue to exercise.** |
|  | Q & A about home exercise | Answer the questions arising from the participants’ home exercise and **provide professional support for them.** |
| 6^th^, 10^th^, 14^th^, 18^th^, 22^th^ week | One-on-one consultation | Through WeChat or telephone, to understand the participants home exercise, **solve the obstacles encountered during the exercise, and encourage them to continue to exercise.** |
